# Evaluation of the Effect of Hyaluronic Acid Injection on the Reconstruction of Reduced Interdental Papillae in Patients Referred to Shiraz School of Dentistry

**DOI:** 10.30476/dentjods.2022.94766.1808

**Published:** 2023-09

**Authors:** Reihaneh Ebrahimi, Hooman Khorshidi, Rabieh Boroumand, Ali Azadikhah, Pardis Haddadi, DMD, MScD

**Affiliations:** 1 Dept. of Periodontology, School of Dentistry, Shiraz University of Medical Sciences, Shiraz, Iran; 2 Dept. of Maxillofacial Radiology, School of Dentistry, Shiraz University of Medical Sciences, Shiraz, Iran

**Keywords:** Aesthetics, Gingiva, Hyaluronic acid

## Abstract

**Statement of the Problem:**

The occurrence of papillary defects adjacent to teeth or dental implants causes both the dental staff and the patients to be concerned about the esthetic issues. Interdental papilla reconstruction surgery is one of the most difficult and unpredictable mucogingival surgeries.

**Purpose:**

The present study aimed to investigate the efficacy of hyaluronic acid injection in the reconstruction of the interdental papilla.

**Materials and Method:**

This clinical trial study was conducted on four patients with 20 deficient interdental papillae who met the inclusion criteria. At first, local anesthesia was applied. Afterward, 0.2 mL of 1.6% hyaluronic acid (HA) gel was injected (at the tip of the papilla and 2-3 mm below the tip of the papilla) three times every two weeks. At baseline, three, and six months later, clinical photography was taken under standard conditions. The papilla height (the distance between the interdental papilla tip and the basis), black triangle area, and the distance between the interdental papilla tip and contact point of adjacent teeth were all measured using Image J software.

**Results:**

The effectiveness of using HA gel in reducing the black triangle area was 85.06%. Furthermore, the papilla length increased by 70.256% while contact to papilla distance decreased by 83.026%. At different times, the values of the studied variables in the three levels were significantly different (*p*< 0.05).

**Conclusion:**

Injection of HA with 1.6% concentration at two points of the interdental papilla was effective in interdental papilla reconstruction at the aesthetic zone, especially in long-term, follow-ups (especially 6 months).

## Introduction

The interdental papilla is a small part of the free gingiva that fills in the space between the teeth, apical to their contact area [ 1 ]. Although it comprises a small percentage of the visible oral soft tissue, it has a unique morphology, histology, and molecular structure. Besides, the interdental papilla serves an important aesthetic function in the anterior maxillae, as its absence leaves a small visible black triangle between the adjunct teeth [ 2 - 3 ]. Food impaction, speaking difficulties, tooth sensitivity, root caries, abrasion, erosion, and plaque accumulation are all consequences of this deficiency [ 1 - 3 ]. As the number of patients requesting cosmetic dental treatments increases, dentists are being compelled to pay closer attention to the visual harmony of gingival topography [ 4 ].

The etiology of interdental papilla deficiency is multifactorial. Gingival recession, a prevalent periodontal problem that results in the apical displacement of the gingival margin and subsequent exposing the root surface to the oral cavity, is one of the most common causes [ 5 - 6 ]. Gingival recession may occur for a variety of reasons, the most common of which is dental plaque [ 7 ]. It can also be caused by rough brushing, dental malposition, bone dehiscence, root alignment and angulation, strong muscular attachment, frenum tension, smoking, and trauma from occlusion [ 8 ]. Some iatrogenic factors, such as some orthodontic and prosthetic treatments, may also contribute to gingival recession [ 9 - 10 ]. Interdental papilla deficiency can be caused by periodontal surgery in case of oral soft tissue contraction during the healing phase [ 11 ].

In order to reconstruct a reduced interdental papilla, dentists should first eliminate the etiological factors before using a combination of different surgical procedures, as none of them provides ideal results when used alone [ 12 ]. Nonetheless, surgical reconstruction of interdental papilla remains one the most difficult and unpredictable aesthetic procedures. Factors such as interdental bone loss or interdental contact change can exacerbate the situation by causing a loss or deficiency in dental papilla height [ 12 ]. Moreover, since there is no reliable blood source, using small-sized bone/gingival grafts may lead to unpredictable results [ 12 ]. Consequently, non-surgical procedures such as orthodontics or restorative procedures might be required following this surgery [ 13 ].

Although largely ignored, less invasive methods such as the use of hyaluronic acid (HA) are present for the reconstruction of the interdental papilla. HA is a linear polysaccharide found the extra-cellular matrix of the connective tissue, also known as synovial liquid. Due to its physiology and structure, it significantly contributes to the uniformity of tissues and has some antibacterial and anti-inflammatory characteristics [ 14 ]. Additionally, HA has numerous therapeutic applications. For instance, it is applied to surgical sites to prevent scar formation [ 15 ]. It is frequently used in orthopedics to treat osteoarthritis and rheumatoid arthritis [ 16 ]. Besides, many studies on this material have been conducted in the field of tissue engineering, as it plays an important role in cell migration, organogenesis, and development [ 17 ]. In dermatology, HA has also been utilized as a filler in dermatology to restore the lost tissue mass [ 18 ]. According to a review of the dental literature, applying HA could decrease localized bleeding during probing and reduce probing depth in patients with gingivitis and periodontitis [ 19 - 20 ]. Moreover, using HA with 1300 K-Dalton weight in guided tissue regeneration (GTR) surgeries may reduce bacterial contamination. It is worth mentioning that this substance elicited no immune response upon direct contact with bone or soft tissue [ 21 - 22 ]. A few recent studies have also shown promising results in terms of utilizing HA injection for the interdental papilla reconstruction [ 2 , 23 - 24 ]. These studies, however, only reported on the use of HA in a single injection site. In addition, they mostly used low-viscosity HA, which was thought to have contributed to the relapses observed during the follow-up sessions. Therefore, the present study aims to investigate the efficacy of high-viscosity HA injection in interdental papilla reconstruction when applied both apically and coronally to the papilla base (the assumed line between the gingival zenith of adjacent teeth).

## Materials and Method

This clinical trial was conducted at the School of Dentistry, Shiraz University of Medical Sciences (Shiraz, Iran) from February to September 2020. The study was approved by the Ethics Committee of Shiraz University of Medical Sciences (Code: IR. SUMS.1399. 030) and registered in the Iranian Registry of Clinical Trials (IRCT20200429047132N1). Patients with interdental papilla loss in the anterior region of jaws (classes I and II Nordland and Tarnow), no gingivitis or periodontitis, and a plaque index of less than 20% were carefully selected from those referred to the dental clinic of the School of Dentistry for deficient interdental papilla treatment. Before beginning the proposed treatment modality, the patients were informed of its potential risks and benefits, alternative surgical treatments, as well as their potential risks and benefits. Finally, written informed consents were obtained from the patients who were willing to participate in the study.

### Sample size and recruitment strategy

The sample size was calculated using data from previous studies on the effectiveness of HA injection in interdental papilla reconstruction. Accordingly, HA was injected into 20 areas in four patients, who had interdental papilla deficiency. Loss of interdental papilla in the anterior region of jaws (classes I and II Nordland and Tarnow), absence of gingivitis and periodontitis, and a plaque index of less than 20% have been defined as the inclusion criteria. Additionally, periodontal considerations included no active periodontal disease, no bleeding on probing, no pocket depth greater than 5 mm, and no signs of tooth mobility. The excluding criteria were smoking, having any systemic disease that might affect the periodontal health or scar healing, taking any medications that could cause gingival enlargement, having teeth crowding, caries, calculus, or wearing orthodontic appliance, history of allergy to injectable fillers, pregnancy and breastfeeding, and history of periodontal surgery in the past 12 months.

### HA injection procedure

**Figure 1:**
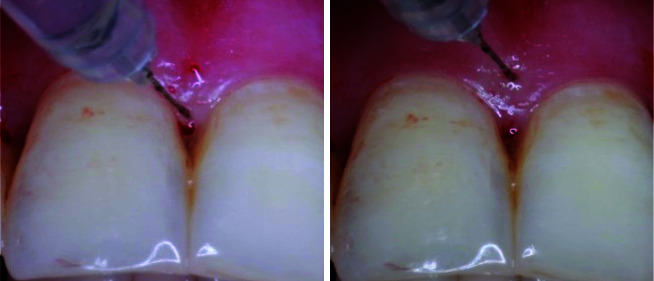
Sites of hyaluronic acid (HA) injection

The treatment procedures were performed by an experienced third-year periodontology resident. The patients were first given local anesthesia (lidocaine 2% + 1:100000 epinephrine). Then, 0.2 mL of HA gel (Synovial Forte 1.6% 32mg, IBSA FARMACEUTICI, Italy, 2020) was administered perpendicular to the injection sites with a 31G-0.3 ml insulin syringe. For each papilla, two injection sites were selected, one located 2-3 mm below the tip of the papilla and the other on the interdental tip [ 24 ]. The injection was continued until the papilla became ischemic. Then, these sites were massaged towards the incisal edge to make the enlargement of the papilla just like a real embrasure. This procedure was repeated three times at two-week intervals [ 24 ] (Figure 1). 

On the day of injection, the patients were instructed not brushing or flossing the region of injection. They were allowed to brush the coronal area of the gingiva the next day, but flossing was not permitted until two weeks after the last injection.

### Data collection

After each session, as well as three- and six-month follow-ups, standard clinical photographs were taken. A Canon E850D digital camera was used, and all photographs featured the University of Michigan O probe with Williams marking for calibration and upcoming comparisons. All photos were taken by one person under the same quality and quantity of light, and a lip retractor was used to homogenize the light. The camera lens was placed 30 cm away from the patient's chin, while he/she sat upright and looked forward. The patient’s Frankfort plane was parallel with the ground along with the lens of the camera. The photos were finally uploaded in the Image J software.

The papilla height (the distance between the papilla’s base and tip), the distance between the apical portion of the interdental contact area and the tip of the papilla, and the black triangle area were measured before starting the treatment, as well as the second and third injection sessions, and during the follow-up sessions [ 24 ]. Using the following formula [ 23 ], the Image J software was used to assess the changes based on the pixel size: 

$n=\mathrm{\%change}\frac{S_{\mathrm{in}}-S_{\mathrm{fin}}}{S_{\mathrm{in}}}=1-{\left(\frac{l_{\mathrm{in}}}{l_{\mathrm{fin}}}\right)}^{2}\frac{N_{\mathrm{fin}}}{N_{\mathrm{in}}}$

The variables are defined as S initial = the area of the gap before the treatment, S final = the area of the gap after the treatment, N initial = the number of gap image pixels before the treatment, N final = the number of gap image pixels after the treatment, L initial = the size of the reference object on the image before the treatment, and L final = the size of the reference object on the image after the treatment.

### Statistical analysis

Data were analyzed using SPSS version 24.0 (IBM, Armonk, NY, USA). Descriptive statistics, including central and dispersion indicators, were obtained. Then, the effectiveness of HA injection in the improvement of the interdental papilla was determined using inferential statistics such as repeated measures tests and multiple comparisons. A value of *p*< 0.05 was considered statistically significant.

## Results

The purpose of this study was to determine the effectiveness of HA injection in the reconstruction of interdental papilla when applied both 2-3 mm below the tip of the papilla and tip of the papilla. The results of the descriptive analysis of the data including the minimum, maximum, mean, and standard deviation of the measurements taken on the 20 injection locations are presented in Table 1. As indicated in the table, the success rate of the studied method in reducing the black triangle area was 85.06%. In addition, the papilla height increased by 70.256%, while the distance between the apical points of interdental contact to the tip of the papilla decreased by 83.026%.

**Table 1:** The descriptive analysis of the data (number of injection areas= 20)

Level	Session	Minimum	Maximum	Mean±SD
Black triangle area	Initial	0.46	5.92	2.09±1.22
	Second	0.06	2.12	1.17±0.60
	Third	0.02	1.84	0.81±0.50
	Three months	0.00	1.68	0.54±0.45
	Six months	0.00	1.32	0.38±0.41
Papilla’s height	Initial	0.82	3.86	2.52±0.82
	Second	1.50	4.59	3.20±0.75
	Third	1.23	4.85	3.53±0.89
	Three months	1.44	5.00	3.65±1.01
	Six months	2.26	5.38	3.96±0.84
Contact to papilla*	Initial	0.73	3.21	1.94±0.65
	Second	0.30	2.35	1.29±0.50
	Third	0.30	2.52	0.98±0.54
	Three months	0.00	1.58	0.57±0.33
	Six months	0.00	0.98	0.36±0.29

* : Distance between the apical point of contact to the tip of the papilla

### Inferential analysis

In order to investigate the effectiveness of the proposed treatment, repeated measurement tests and multiple comparisons were used. The black triangle area, papilla height, and distance between the apical point of contact to the tip of papilla were all significantly different at all three levels, including before the treatment and after the second and third treatment sessions (*p*< 0.05). 

**Figure 2:**
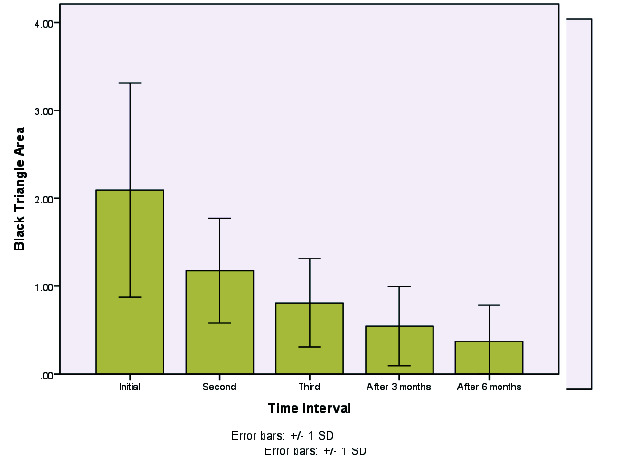
Mean change of the black triangle area

**Figure 3:**
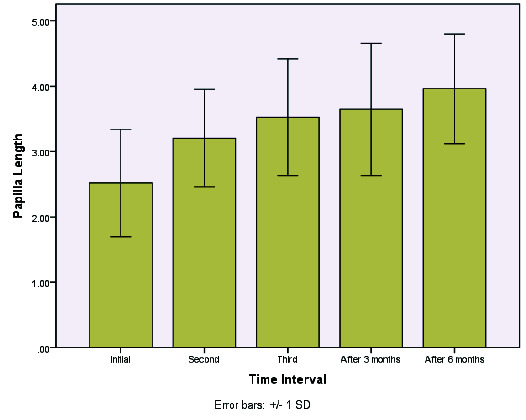
Mean change of the length of the papilla

**Figure 4:**
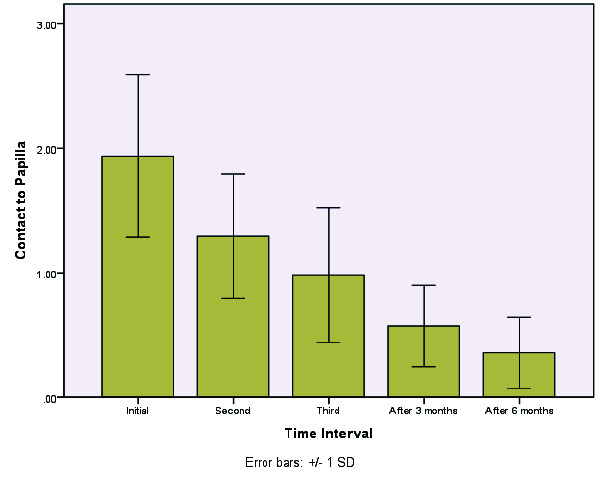
Mean change of the contact point to the tip of the papilla

**Figure 5:**
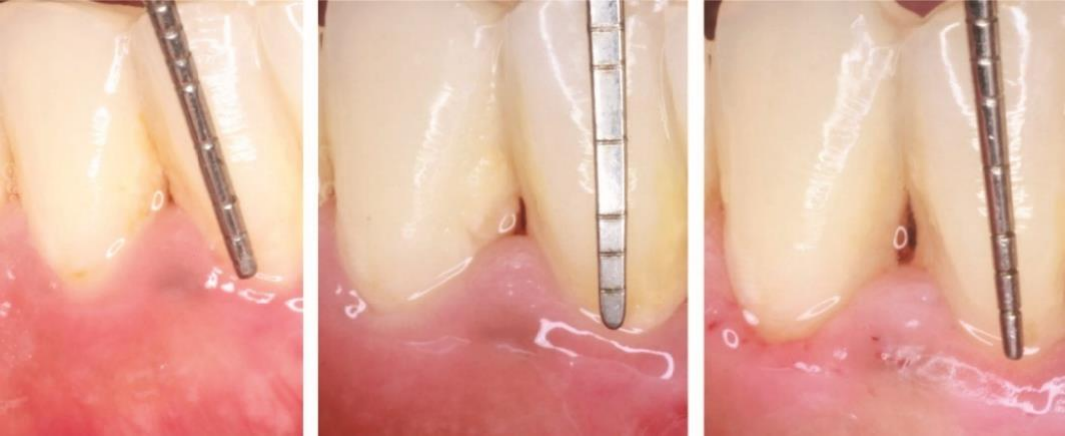
before injection, after three-month follow-up, and six-month follow-up

Multiple comparisons (Figures 2-5) revealed a significant difference in the levels of the black triangle area and contact with papilla during all sessions (*p*< 0.005). Given the negative values of the mean differences, it could be concluded that the values of the studied variables decreased significantly over time. Moreover, considering the papilla height, all the comparisons (except for the third injection time and the first follow-up) were statistically significant (*p*< 0.05). Accordingly, a significant difference was observed in the values of this variable as the treatment sessions went on. Given the positive mean differences, it could be concluded that the values of the variables increased significantly over time, particularly in long-term follow-ups after six months. 

## Discussion

A review of the existing literature revealed that few studies have been done on the use of HA in papillary reconstruction [ 1 - 2 , 23 - 24 ]. The present study was the first to examine HA at a greater concentration (1.6%) injected into two interdental papilla areas (2mm be low the tip of the papilla and the papilla tip). The results indicated statistical and clinical improvements at three-month and six-month follow-ups. Accordingly, using HA gel was successful in reconstructing the interdental papillary deficiencies and reducing the black triangle area. During the six-month follow-up, the papillary deficiencies improved by 85.06%. Similarly, Becker *et al*. [ 23 ] reported 94% improvement in papillary deficiencies and 76% improvement in areas adjacent to implants. Moreover, Sadat Mansouri *et al*. [ 24 ] observed 22-100% improvement in papillary deficiencies. Awartani *et al*. [ 2 ] also found a 41-62% reduction in the interdental black triangle area, and Lee *et al*. [ 1 ] showed 92.55% success in interdental papilla reconstruction in the anterior maxillary and mandibular areas. In the same line, Tanwar *et al*. [ 25 ] conducted a case report study and only used one interdental site to inject HA gel. In the three-month follow-up, the results indicated that the reconstruction of the lost papillae was satisfactory. Overall, these studies demonstrated that the injection of HA gel, as a safe substance, significantly reduced the area of the interdental black triangles, which was consistent with the findings of the current study.

The results of the present study indicated that the papillary defects improved between the injection sessions, which were similar to the findings of Becker *et al*. [ 23 ]. However, no significant improvement was detected in this regard between the third injection session and the first follow-up session (after three months). Meanwhile, in comparison to the baseline measurements, the differences seen in the three-month and six-month follow-ups were significant. In the study by Awartani *et al*. [ 2 ], the improvement of papillary defects between the injection sessions was not statistically significant. No information was provided in other studies concerning the improvements between the injection sessions. Nonetheless, all of the previous investigations, like the current one, indicated a decrease in the area of the interdental papillary defects (black triangles) over time [ 1 , 24 ]. 

In the current clinical trial study, all patients received HA gel injections in three sessions at two-week intervals, with two injection sites at 2-3 mm below the tip of the papilla and the tip of the papilla. It is noteworthy that the number of HA gel injections was not the same in the previous studies conducted by Becker *et al*. [ 23 ], Lee *et al*. [ 1 ], and Sadat Mansouri *et al*. [ 24 ]. Becker *et al*. [ 23 ] administered injections twice at eight sites and three times at 12 sites. On the other hand, Sadat Mansouri *et al*. [ 24 ] performed injections at most three times if the black triangles were not eliminated during the treatment sessions. The number of injections administered in the study by Lee *et al*. [ 1 ] varied depending on the severity of the papillary defects. This could be one of the reasons for the disparities in the findings of these studies.

In the current investigation, three-month and six-month follow-ups were considered, and significant and desirable results were observed during the long-term follow-ups. These results were in agreement with those reported by Sadat Mansouri *et al*. [ 24 ] (with three-month and six-month follow-ups) and Awartani *et al*. [ 2 ] (with four-month and six-month follow-ups). In the study by Becker *et al*. [ 23 ], if the patients were cooperative enough, the follow-ups were even continued up to 25 months.

The findings of the present study indicated that the treatment outcomes did not regress over time, as was observed in the follow-ups. This was consistent with the findings of the research by Sadat Mansouri *et al*. [ 24 ]; however, Awartani *et al*. [ 2 ] reported that treatment outcomes regressed over time (between the four-month and six-month follow-ups). Accordingly, larger black triangle areas were found in the six-month follow-up than in the four-month follow-up. The brand of the applied HA gel and the dimensions of the treated papillae were mentioned as the probable reasons for these findings. In a study by Lee *et al*. [ 1 ], the areas of the interdental papilla that were completely filled after injection (areas in which black triangles were completely eliminated) remained the same in the six-month follow-up, while some relapses were found in the areas of the interdental papilla that were not fully healed [ 1 ]. The differences in the success rate reported in the current study versus those reported by Becker *et al*. [ 23 ], Lee *et al*. [ 1 ], and Sadat Mansouri *et al*. [ 24 ] could be attributed to the critical role of the anatomy of the region, the morphology of the papillae, differences in the initial severity of papillary defects, the number of injection sites in each papilla, the injection protocol, the number of injections, the viscosity and brand of the applied gel, and follow-up times.

Some of the limitations of the current research were the small statistical population of the patients and the regions under investigation, as well as short follow-up times. Another limitation of the study was the use of two-dimensional analyses to examine the three-dimensional mass changes in the interdental papilla or the areas adjacent to implants. On the other hand, this trial had several strong points, including the use of HA gel with 1.6% concentration, the performance of three regular injections at two sites for all the studied interdental papillae at two-week intervals and the patients' follow-up for up to six months.

Further research with larger statistical populations is recommended to evaluate the improvements of papillary defects using three-dimensional analyses. Additionally, considering the increased usage of dental implants and the challenges involved in anterior papillary reconstruction, it is essential to design a study to investigate the success rate of HA gel injection in implant-adjacent areas.

## Conclusion

Injection of 1.6% viscosity HA gel at two points of the interdental papilla was effective in reconstructing the interdental papilla at the aesthetic zone, especially in long-term follow-ups (six months). Thus far, further studies with different HA gel viscosities, larger sample sizes, and longer follow-ups are required to investigate the long-term relapses and the necessity of retreatment.

## Acknowledgment

The authors would like to thank Dr. Emami for her kind contribution and generous support to the completion of this work. 

## Conflict of Interest

The authors declare that they have no conflict of interest.
